# Possible roles of anti-type II collagen antibody and innate immunity in the development and progression of diabetic retinopathy

**DOI:** 10.1007/s00417-021-05342-6

**Published:** 2021-08-11

**Authors:** Tsunehiko Ikeda, Kimitoshi Nakamura, Teruyo Kida, Hidehiro Oku

**Affiliations:** 1 Department of Ophthalmology, Osaka Medical and Pharmaceutical University, Takatsuki City, Osaka, Japan; 2 Department of Ophthalmology, Osaka Kaisei Hospital, 1-6-10 Miyahara Yodogawa-ku, Osaka City, Osaka, Japan; 3 Nakamura Eye Clinic, Matsumoto City, Nagano, Japan

**Keywords:** Diabetic retinopathy (DR), Rheumatoid arthritis (RA), Type II collagen, Autoimmunity, Innate immunity, NOD-like receptor family pyrin domain-containing 3 (NLRP3), Pyroptosis, Efferocytosis, Specialized pro-resolving mediators (SPMs)

## Abstract

The pathogenesis of both diabetic retinopathy (DR) and rheumatoid arthritis (RA) has recently been considered to involve autoimmunity. Serum and synovial fluid levels of anti-type II collagen antibodies increase early after the onset of RA, thus inducing immune responses and subsequent hydrarthrosis and angiogenesis, which resemble diabetic macular edema and proliferative DR (PDR), respectively. We previously reported that DR is also associated with increased serum levels of anti-type II collagen antibodies. Retinal hypoxia in DR may induce pericytes to express type II collagen, resulting in autoantibody production against type II collagen. As the result of blood-retinal barrier disruption, anti-type II collagen antibodies in the serum come into contact with type II collagen around the retinal vessels. A continued loss of pericytes and type II collagen around the retinal vessels may result in a shift of the immune reaction site from the retina to the vitreous. It has been reported that anti-inflammatory M2 macrophages increased in the vitreous of PDR patients, accompanied by the activation of the NOD-like receptor family pyrin domain-containing 3 (NLRP3) inflammasome, a key regulator of innate immunity. M2 macrophages promote angiogenesis and fibrosis, which might be exacerbated and prolonged by dysregulated innate immunity. 
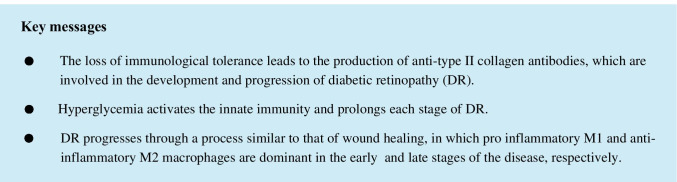

## Introduction

It is generally accepted that diabetic retinopathy (DR) is one of the chronic inflammatory diseases [1]. The clinical findings of DR include (1) increased blood levels of inflammatory biomarkers, such as C-reactive protein, fibrinogen, and neutrophil count [2–4]; (2) increased vitreous levels of inflammatory cytokines, such as interleukin (IL)-1β, tumor necrosis factor-alpha (TNF-α), and IL-6 [5, 6]; 3) infiltration of immune cells, such as macrophages, lymphocytes, and neutrophils, in the epiretinal and internal limiting membranes obtained during vitrectomy [7, 8]; (4) increased expressions of leukocyte adhesion molecules in the blood vessels of the retina and choroid [9]; (5) neutrophil entrapment in the retinal microcirculation [10]; (6) neutrophil infiltration in the choroidal capillaries [11]; and (7) activation of the renin-angiotensin system that enhances chronic inflammation [12]. These findings indicate that DR has a chronic inflammatory etiology. In addition to chronic inflammation, the involvement of autoimmunity in the etiology of DR has recently attracted considerable attention [13, 14]. It has been reported that HLA-DR and HLA-DQ antigens, types of HLA class II molecules, are related to the development and progression of DR [15, 16]. The presence of autoantibodies in the serum of DR patients [14, 17–22] and the effectiveness of immunosuppressants, such as methotrexate, sirolimus (rapamycin), cyclosporin A, TNF-α inhibitors, and corticosteroids in treating diabetic macular edema (DME) [23–27], may also indicate the possibility that DR arises from autoimmunity.

In spite of poor glycemic control, diabetic patients who do not always develop DR after a long duration of the disease and patients with non-proliferative DR (NPDR) do not necessarily progress to proliferative diabetic retinopathy (PDR). Although many other factors, such as genetics, retinal ischemia and comorbidities, and myopia, may contribute to the onset and progression of DR, the individual differences may be due to the interplay of the various pathophysiological factors, including immune response.

We measured anti-type II collagen antibodies in the serum of DR patients and found that they were higher compared with the non-diabetic control group [20]. Based on the results of that study and a review of the previously published literature, we wish to herein discuss the likely role of immune response in the development of DR.

## Striking similarities between diabetic retinopathy and rheumatoid arthritis

Rheumatoid arthritis (RA) is a typical disease with chronic inflammatory and autoimmune features [28]. The pathological conditions of RA are characterized by chronic inflammation of the joint associated with angiogenesis and fibroblast proliferation [29]. Similar to the vitreous body, type II collagen and hyaluronic acid are abundant in the articular cartilage and joint space, respectively [30]. Autoimmune reactions to type II collagen have been shown to be involved in the pathogenesis of RA [31, 32], where Arthus reaction, a type of local type III hypersensitivity, occurs in the joint [33, 34], thus causing inflammation and destruction of the articular cartilage [28]. Persistent chronic inflammation of the joint causes hypoxia of synovial cells lining the inner surface of the joint capsule as well as angiogenesis induced by vascular endothelial growth factor (VEGF) and proliferation of synovial cells [34, 35]. As a result, fibrovascular tissues called “pannus” are formed in the joint [36]. Similar findings to the advanced stage of RA are present in PDR patients, including retinal hypoxia, VEGF-induced angiogenesis, and proliferation of glial cells [37], resulting in the formation of proliferative membranes in the vitreous and vitreoretinal interface [38].

**Fig. 1:**
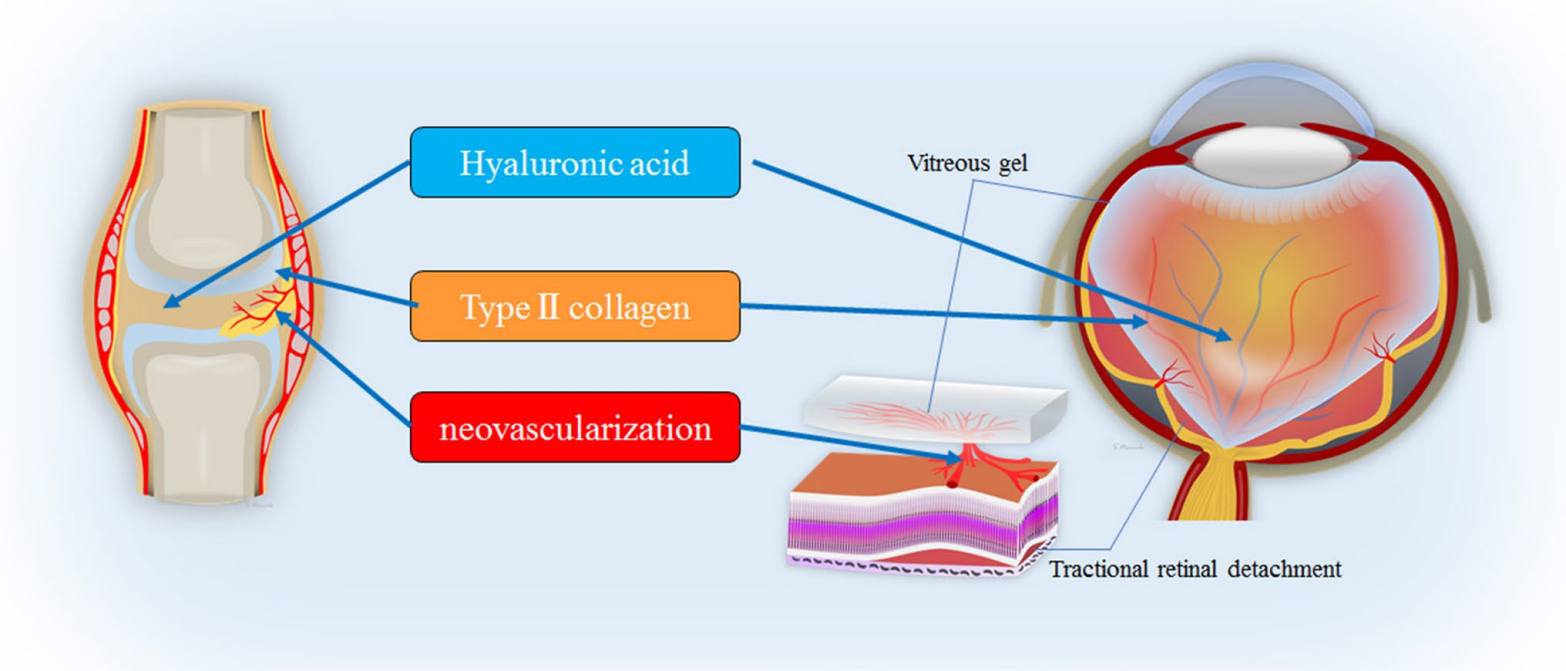
Schematic images showing that anatomical structure and macromolecular composition of the joint and vitreous body. These images illustrate the pathophysiological similarity between the advanced stage of rheumatoid arthritis (RA) and proliferative diabetic retinopathy (PDR)

In this current review, we focused on the similarity of the anatomical structure and macromolecular composition between the vitreous body and the joint and the pathophysiological similarity between DR and RA (Fig. 1).

## Cartilage-affecting diseases and anti-type II collagen antibody

The assumption that the autoimmune responses to type II collagen may be involved in the pathogenesis of RA is supported by increased serum and synovial fluid levels of anti-type II collagen antibodies in the early stage of RA [32, 39] and by the observation that immunizing animals with type II collagen results in the formation of RA-like joint lesions [40, 41]. As type II collagen-containing tissues such as the articular cartilage and vitreous body are avascular, type II collagen is considered to be a sequestered antigen that can escape immune surveillance, resulting in immunological tolerance [42, 43]. Autoantibodies to type II collagen will be formed by the loss of immunological tolerance in RA patients, causing the progression of autoimmune-mediated joint destruction [43].

Increased serum levels of anti-type II collagen antibodies have been observed in other diseases affecting cartilaginous tissues of the joints (e.g., osteoarthritis, relapsing polychondritis, systemic lupus erythematosus, chronic gouty arthritis, and temporomandibular joint disturbance syndrome) [31, 44–47]. Besides serum anti-type II collagen antibodies and animal models using type II collagen immunization, the administration of a small amount of undenatured type II collagen reportedly induces oral immune tolerance to ameliorate the symptoms of RA, as described later in detail [48–50]. Altogether, this evidence suggests that anti-type II collagen antibodies might have a causative role as opposed to being a bystander of the diseases. Cartilaginous tissues are also present in the inner ear (and a part of auditory ossicles), and increased serum levels of anti-type II collagen antibodies have also been detected in the diseases affecting the inner ear, such as Meniere’s disease, autoimmune ear disease (AIED), and otosclerosis [51–53]. The administration of type II collagen has been shown to cause Meniere’s disease- and AIED-like conditions in animals [54, 55], thus raising the possibility that autoimmunity to type II collagen may be involved in the development of these diseases that affect the inner ear.

## Diabetic retinopathy and anti-type II collagen antibody

**Fig. 2:**
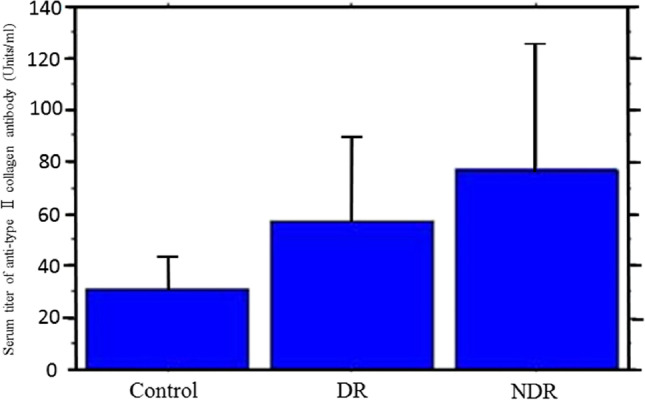
This graph shows the serum levels of anti-type II collagen antibodies (units/ml) in diabetic retinopathy (DR) patients. The serum levels of anti-type II collagen antibodies in DR patients are significantly higher than those in control subjects. Notably, the levels are higher in the group of patients with non-diabetic DR than in the group of patients who already had DR

Balashova et al. firstly reported that increased levels of anti-type II collagen antibodies and immune complexes were observed in the serum and lacrimal fluid of DR patients [19]. We also measured the serum levels of anti-type II collagen antibodies in DR patients and found significantly higher levels of autoantibodies to type II collagen in DR patients compared to control subjects (Fig. 2) [20]. Remarkably, the serum levels of anti-type II collagen antibodies were higher in patients without DR than in patients with DR [20]. These results suggest that anti-type II collagen antibodies, which already increase in serum before the symptoms of DR are manifested, may be one of the factors involved in the onset of DR.

As mentioned above, the vitreous body, along with the joint and inner ear, is one of the few tissues that contain type II collagen, a component of cartilage. Since it has been considered that the autoimmunity to type II collagen is a causative factor in the pathogenesis of the cartilage-affecting diseases, such as RA and Meniere’s disease, we assume that the similar mechanism may be involved in the development and progression of DR, a disease affecting the vitreous body in which type II collagen is abundantly present.

Although the mechanism by which the serum levels of anti-type II collagen antibodies increase in diabetic patients remains unclear, it has been shown that hypoxia and hyperglycemia caused blood-ocular barrier breakdown in those patients [56, 57], which may induce macrophages to migrate into the ocular tissues and to phagocytize type II collagen that is normally sequestered from the immune system. The peptide fragments of type II collagen presented by macrophages can be recognized by helper T cells, resulting in the production of anti-type II collagen antibodies by activated B cells [58]. Since activated microglia are capable of phagocytosis and antigen presentation, the involvement of retinal microglia in anti-type II collagen antibody production is undeniable [59, 60].

## Proposal of an anti-type II collagen antibody-associated disease

The vitreous body, joint, and inner ear, all of which contain type II collagen, are filled with liquid, namely the vitreous humor, synovial fluid, and endolymph, respectively. It is interesting to note that all of these tissues have also barrier mechanisms to the blood, i.e., the blood-ocular barrier, blood-joint barrier, and blood-labyrinth barrier, respectively [61–63], which separate type II collagen in these tissues from circulating immune cells. It seems likely that the disruption of these barriers results in the loss of immunological tolerance to type II collagen and the subsequent development of DME in DR, hydrarthrosis in RA, and endolymphatic hydrops in Meniere’s disease [64, 65]. We have recently reported that the serum levels of anti-type II collagen IgG antibody were significantly higher in patients with epiretinal membrane than in control patients [66]. Consequently, chronic disorders affecting the vitreous body and cartilaginous tissues, including the above three diseases, are supposed to be in the same spectrum of immune tolerance breakdown to type II collagen, causing anti-type II collagen antibody production.

## Immunological divergence between diabetic retinopathy and rheumatoid arthritis

Despite the speculated involvement of immune responses to type II collagen in the pathogenesis of both DR and RA, it seems rather uncommon that these two diseases develop simultaneously. In fact, it was previously reported that patients with RA were less predisposed to develop DR [67]. A number of studies have indicated the frequency of HLA-DR and -DQ antigens that were associated with disease susceptibility were different between DR and RA patients [15, 16, 68]. For example, HLA-DRB1*0401 and *0405 alleles are reportedly strongly associated with RA susceptibility [69–72], while these single alleles are non-susceptible to DR. Conversely, it has been shown that the HLA-DRB1*0402 allele was associated with resistance to developing RA [73, 74], although a strong positive correlation between B1*0402 and DR susceptibility were reported [75].

A possible explanation for that finding is that there may be structural differences of collagen molecules recognized by lymphocytes between these two diseases. It has been reported that autoantibodies against citrullinated type II collagen were produced in RA patients [76, 77]. Meanwhile, collagens including type II are reportedly glucosylated in diabetic patients [78, 79]. Bassiouny proposed that glucosylated collagen may increase antigenicity to initiate autoimmune responses leading to diabetic complications, presumably indicating that T cells recognize glycosylated type II collagen as “not self” [80]. Although autoantibodies to native type II collagen are also found in the serum of DR and RA patients [19, 20, 81, 82], autoimmune reactions to modified type II collagen seem to be critical for the onset of these diseases, because the coincidence of DR and RA is uncommon as mentioned above.

Conversely, Mimura et al. reported that the frequency of the HLA-DR4-DQ4 haplotype, which was associated with RA, especially in more severe cases [83, 84], was significantly higher in PDR patients than in a non-DR group [85, 86]. HLA-DR and HL-DQ are class II major histocompatibility complex (MHC) antigens, expressed on the surface of antigen-presenting cells, such as macrophages, dendritic cells, and B cells, and determine the productivity of the specific antibodies against proteins. Thus, it is therefore possible that HLA-DR4-DQ4, which are frequently observed in patients with progressed stages of DR and RA, might be involved in the production of some specific autoantibodies. Banerjee et al. found that high levels of anti-native type II collagen antibodies in the serum of RA patients were associated with HLA-DR4 [82]. Matsushita et al. reported that 94 putative DQ4-binding motifs (i.e., amino acid sequences) were detected in the native type II collagen molecules [83]. Cook et al. observed that the presence of antibodies to native type II collagen was associated with the activity of RA and severity of symptoms [32, 45]. As described above, a considerable number of HLA-DQ4-binding motifs were reportedly found in native type II collagen [83], presumably implying that immune response to native type II collagen could be involved in the progression of DR and RA.

## Diabetic retinopathy and Arthus reaction

The joint lesions of RA have been considered to be caused by Arthus reaction, as evidenced by neutrophil infiltration, increased serum and synovial fluid levels of complements and immune complexes, and tissue deposition of immune complexes [33, 87, 88].

The pathological features of Arthus reaction are bleeding, thrombosis, and edema [89], then followed by fibrinoid deposition, as observed in the RA joint [90]. Fibrinoid is mainly composed of fibrin and immune complexes [91], of which deposition has reportedly been detected in the joint tissues of RA patients [92].

We assume that DR in the early stage may also have pathological features of Arthus-like reaction as following reasons: (1) neutrophil infiltration into the retinal tissue [10], (2) increased serum levels of complements and immune complexes [19, 93], (3) deposition of immunoglobulins and complements, components of immune complexes, in the retina [94, 95]. Fu et al. observed that co-staining for oxidized low-density lipoprotein (oxLDL) and IgG was present in the diabetic retina, presumably indicating the deposition of anti-oxLDL immune complexes [96]. Giusti proposed that immune complex deposition in the retina was implicated in the pathogenesis of DR [97]. Retinal bleeding, thrombosis, and edema, all of which are symptoms of Arthus reaction [87], are frequently observed in the relatively early stage of DR [98].

It is sometimes described that hard exudates consist of lipids and/or lipoproteins [99]. However, proteins including fibrin were also reportedly present in hard exudates [100]. Liu et al. demonstrated that lipoprotein (a) [Lp(a)] bound covalently to fibrin, contributing to the deposition of Lp(a), colocalized with fibrin in atheroma [101]. Smith insisted that fibrin was a factor in lipid accumulation in the atherosclerotic plaque, because fibrin is bound to Lp(a) with high affinity and also bound to low-density lipoprotein (LDL) [102]. Nogornev proposed that the atherosclerotic plaque was formed by the deposition of immune complexes containing lipoproteins [103]. We assume that fibrin and immune complexes, components of fibrinoid, may form hard exudates along with lipoproteins by a similar mechanism as atheroma plaque formation.

## Pericytes and type II collagen

Pericytes are considered to have mesenchymal stem cell (MSC)-like properties being able to differentiate into chondrocytes, osteoblasts, and adipocytes [104, 105]. Farrington-Rock et al. demonstrated that when cultured at high density in the presence of a defined chondrogenic medium, pericytes expressed mRNA of Sox9, a chondrocyte marker, and type II collagen [106].

Ihanamäki et al. have shown that the expression of Sox9 and type IIA procollagen mRNA increased in the developing and aging retina in mice [107]. Swinscoe et al. reported that type II collagen was a major component of the bovine retinal microvessel extracellular matrix [108]. MSCs reportedly tend to undergo chondrogenesis under hypoxia [109, 110]. Hypoxic chondrocytes are also known to produce an increased amount of type II collagen [111, 112]. Besides, high glucose reportedly induces chondrogenesis in MSCs [113]. Chondrogenic differentiation culture medium contains high concentrations of glucose [114]. Accordingly, MSC-like pericytes around the retinal vessels may produce type II collagen especially under hypoxia and high-glucose conditions in the diabetic retina.

As mentioned previously, type II collagen produced by pericytes in the diabetic retina will be phagocytized by circulating monocytes/macrophages or retinal microglia, simulating B cells to produce anti-type II collagen antibodies [58–60]. Hypoxia and high-glucose conditions of diabetic retina cause disruption of the blood-retinal barrier [56, 57]. As a result, anti-type II collagen antibodies in the serum may come into contact with type II collagen around the retinal vessels, forming immune complex deposition in the retina.

Selective loss of pericytes occurs in the early stage of DR [115]. It has been reported that increased serum levels of anti-pericyte antibodies were observed in DR patients [14, 17, 18]. In addition to autoimmune responses to type II collagen, anti-pericyte antibodies may injure the pericytes, causing a loss of pericytes in the diabetic retina. A continued loss of pericytes and type II collagen around retinal vessels could result in a shift of the immune response site from the retina to the vitreous and vitreoretinal, where type II collagen is abundantly present [116].

**Fig. 3:**
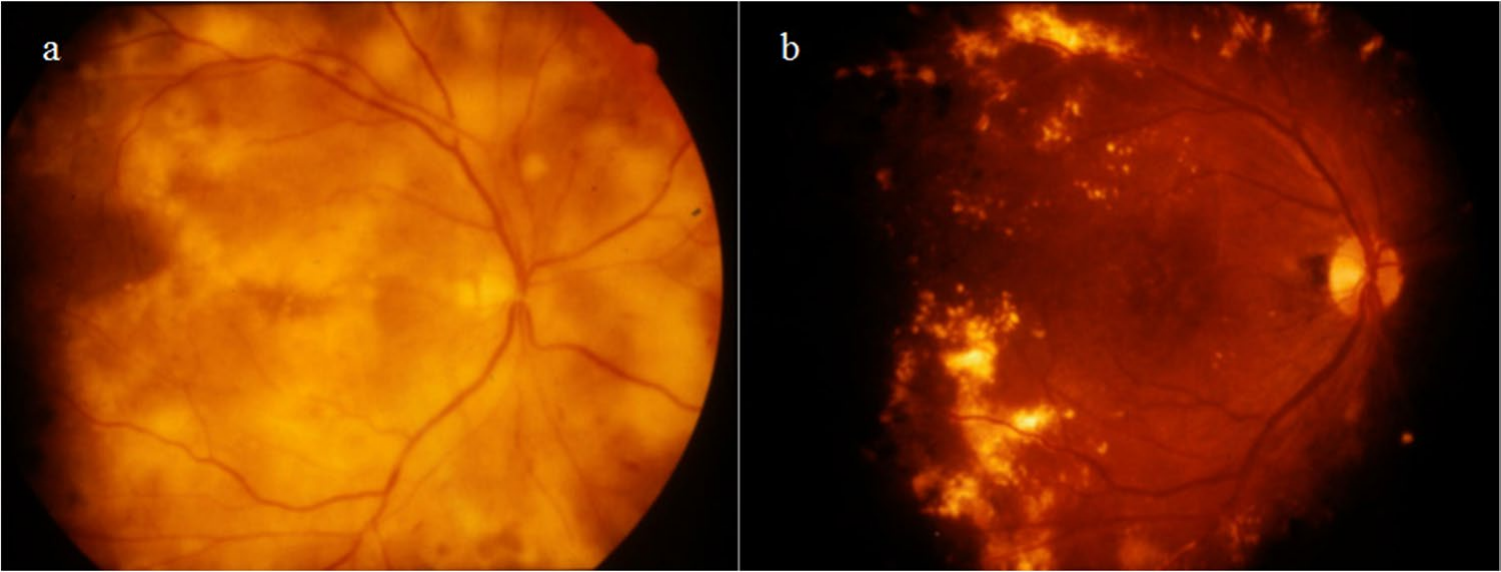
Ocular fundus photographs in a case of diabetic maculopathy with severe hard exudates before vitrectomy (**a**) and after vitrectomy (**b**). The hard exudates in the retina (or in the subretinal space) are gradually reduced only by removing the vitreous body. It is speculated that the improvement of retinal oxygenation suppresses the chondrogenic differentiation of pericytes, resulting in the disappearance of hard exudates containing anti-type II collagen immune complexes

Vitrectomy had been frequently performed to treat DME before anti-VEGF therapy was clinically available [117, 118]. The proposed mechanisms underlying the efficacy of vitrectomy for DME include the elimination of inflammatory cytokines from the vitreous body and the release of vitreoretinal traction [119, 120]. It has also been shown that vitrectomy significantly increased intraocular oxygen tension for prolonged periods after surgery [121]. Stefánsson proposed that vitrectomy improved retinal oxygenation to reduce DME [122]. Hard exudates, which are lesions that are often observed in DME, can be gradually reduced only by removal of the vitreous body (Fig. 3) [117]. According to the aforementioned assumptions, hard exudates might be anti-type II collagen immune complex deposition along with fibrin and lipoproteins. We speculated that the increased retinal oxygen tension after vitrectomy would suppress the chondrogenic differentiation of pericytes producing type II collagen, resulting in the disappearance of hard exudates containing anti-type II collagen immune complexes.

## Müller cells and type II collagen

Müller cells are the predominant glia of the retina with an elongated shape, spanning across the entire retina [123]. Müller cells not only serve as mechanical support of the neural retina, but also play an important role in the maintenance of its metabolic and physiological homeostasis, including regulation of extracellular environment [123, 124], removal of debris [125], and antigen presentation manifested after removal of suppressive activity [126].

Müller cells have been shown to be capable to phagocytize type II collagen [127, 128], which scarcely exists in the retina under physiological conditions except for the perivascular area [109]. Removal of type II collagen by Müller cells may suppress autoimmune reactions against it [127, 128]. On the other hand, the in vitro experiment showed Müller cells synthesized the various collagens of the vitreous and vitreoretinal interface including type II collagen [129]. Müller cells also reportedly expressed the transcription factor Sox9, which directly regulates COl2A1, the gene encoding type II collagen, during development and injury [130]. We tentatively identified immature Müller cells around the foveola, in which GFAP and vimentin were colocalized [131]. We presume that these immature Müller cells might dedifferentiate and produce type II collagen under hypoxia and high glucose conditions in the diabetic retina, forming foveal hard exudates containing anti-type II collagen immune complexes after a loss of pericytes.

As described above, there is some corroborating evidence that type II collagen is involved in the pathogenesis of DR; however, there are presently limitations to making a specific determination.

## Dysregulated innate immunity and multiple autoantibody production in diabetic retinopathy

As described previously, we observed the serum levels of anti-type II collagen antibodies were higher in patients with non-diabetic DR than in patients with DR [20], indicating that humoral immunity to type II collagen might decrease with the progression of DR. Balashova et al. and Danilova et al. found that cellular immunity was suppressed in DR patients [19, 132]. Loukovaara et al. also reported that T cell-mediated responses did not dominate in PDR patients [133]. Meanwhile, Graves et al. and Xu et al. indicated that dysregulated innate immune responses associated with inflammation may contribute to the progression of DR [134, 135]. It has been shown that activated innate immunity promoted angiogenesis and fibrosis [136, 137]. We speculate, therefore, that innate immunity instead of acquired immunity may mainly affect the inflammatory responses as DR progresses.

High levels of autoantibodies to oxLDL and to cardiolipin were observed in the serum of PDR patients [22, 23], although innate immunity seems to be the predominant immune response in PDR as described above. Interestingly, elevated levels of anti-oxLDL and anti-cardiolipin antibodies were also found in the serum of RA patients [138, 139], although anti-oxLDL antibodies in DR patients belonged to the IgA class [21]. These findings also appear to indicate the pathological similarity of DR to RA.

Multiple autoantibodies in the serum are frequently associated with autoimmune diseases, which are caused by a phenomenon called epitope spreading [140–142]. High serum levels of anti-type II collagen antibodies were reportedly associated with the early stage of RA [32, 39], while anti-cyclic citrullinated peptide (anti-CCP) antibodies increased in the late stage [143], indicating that autoantigens involved in the autoimmune diseases may vary with their progression. Complicated immune responses, including activated innate immunity and multiple autoantibody production, may promote the progression of DR.

## Activation of the NLRP3 inflammasome, a critical component of innate immunity, in diabetic retinopathy

NOD-like receptor family pyrin domain-containing 3 (NLRP3) is a component of inflammasome and a key regulator of innate immunity [144]. NLRP3 inflammasome activation leads to caspase-1-dependent production of IL-1β and IL-18 and to pyroptosis (caspase-1-dependent cell death) [145, 146]. Subsequently, the above pro-inflammatory cytokines and damage-associated molecular patterns (DAMPs), such as high-mobility group box 1 **(**HMGB1) and adenosine triphosphate (ATP), are released from pyroptotic cells [147, 148].

HMGB1 binds to its receptors, toll-like receptor (TLR)2, TLR4, TLR9, and RAGE (receptor for advanced glycation end products), then activating NLRP3 inflammasome [149, 150]. Extracellular ATP mediates NLRP3 inflammasome activation through the P2X7 receptor [151]. The NLRP3 inflammasome has reportedly been implicated in the pathogenesis of various diseases, including autoimmune diseases such as RA, inflammatory bowel disease (IBD), multiple sclerosis (MS), and type I diabetes [152–154]. It has been demonstrated that the activation of NLRP3 inflammasome promoted pathological angiogenesis and fibrosis in animal experiments [155, 156].

High glucose and accumulation of ROS (reactive oxygen species) and AGEs (advanced glycation end products) reportedly activate NLRP3 inflammasome [157–159], and these states are observed in the diabetic retina and vitreous body [79, 160, 161]. Increased DAMPs (e.g., HMGB1and ATP) released from necrotic and pyroptotic cells in DR may also activate NLRP3 inflammasome [148, 162, 163]. Chen et al. found that increased gene and protein expression of NLRP3 and caspase-1 was observed in peripheral blood monocytes of DR patients compared with that in normal controls [164]. Charmoy et al. reported that NLRP3 inflammasome mediated neutrophil recruitment [165]. Hao et al. indicated that NLRP3 inflammasome activation increased permeability of the blood-retinal barrier [166]. Therefore, exaggerated inflammatory responses, such as persistent leukocyte infiltration and leakage from the retinal vessels, observed in non-PDR with DME are considered to be at least partly caused by the activation of NLRP3 inflammasome [167, 168].

Loukovaara et al. found that the levels of inflammasome components, including NLRP3 and caspase-1, and inflammasome-related pro-inflammatory cytokines, IL-1β and IL-18, were increased in the vitreous of PDR patients [133]. HMGB1 and extracellular ATP, which activated NLRP3 inflammasome, were also reportedly increased in PDR vitreous [169, 170]. Consequently, innate immunity, in which NLRP3 plays a critical role, might be activated throughout all stages of DR, contributing to exacerbation and prolongation of inflammation.

## Resemblance of diabetic retinopathy to chronic wound healing

We consider that the development and progression of DR may closely resemble a prolonged wound healing process. Wound healing is divided into four phases: hemostasis, inflammation, proliferation, and remodeling [171, 172]. Bleeding and blood clotting are observed in the hemostasis phase [173]. Fibrin is involved in clot formation [174]. The inflammation phase is marked by chemotaxis of immune cells, increased vascular permeability, and removal of cellular debris by macrophages [175–177]. The proliferation phase is characterized by angiogenesis and fibroplasia/fibroblast proliferation [178, 179]. The remodeling phase is where the synthesis of collagen and other extracellular matrix components increases the tensile strength of the wound as it matures [175, 179, 180]. Bleeding, fibrin deposition, and vascular leakage are frequently observed in NPDR [98, 100, 181], and angiogenesis and fibrosis are observed in PDR [182]. We speculate therefore that the former two and the latter two phases of the wound healing process correspond to NPDR and PDR, respectively.

During the inflammation phase of wound healing, pro-inflammatory M1 macrophages exert functions as antigen presentation, phagocytosis, and production of inflammatory cytokines [183, 184]. After that, during the proliferation phase, anti-inflammatory M2 macrophages stimulate angiogenesis and fibroplasia [185–189]. Macrophage polarization similar to the wound healing process has been observed in DR as described as follows.

Omri et al. reported macrophages expressed inducible nitric oxide synthase (iNOS), a marker of M1 macrophage, migrated through the retina in the animal model of early-stage DR [190]. Arroba et al. also found that microglia, the tissue macrophage of the retina, expressed iNOS increased in the animal model of non-PDR [191]. On the other hand, Kobayashi et al. and Abu El-Asrar et al. demonstrated that CD163-positive M2 macrophages were significantly increased in the vitreous of PDR patients [192, 193].

Reportedly, M1 macrophages produce pro-inflammatory cytokines (including TNF-α and IL-6), inducing neutrophil recruitment and vascular permeability [194, 195], while M2 macrophages produce anti-inflammatory cytokines (including IL-10 and TGF-β) and pro-angiogenic factors (including VEGF and PDGF) [196, 197], promoting angiogenesis and fibrosis [198]. Accordingly, the aforementioned skewing of macrophage phenotype may explain well the pathology of wound healing and DR.

## Presumable mechanisms of prolonged inflammatory responses in the diabetic retina

As described above, M2 macrophages that increase in the advanced stage of wound healing release anti-inflammatory cytokines such as IL-10 and TGF-β [196]. M2 macrophages also possess high capacities to produce specialized pro-resolving mediators (SPMs) such as lipoxins, resolvins, protectins, and maresins [199]. SMPs are metabolites of ω3 and ω6 polyunsaturated fatty acids, which play key roles in the resolution of inflammation [200]. As a result, the inflammation and proliferation phases of physiological wound healing cease within a relatively short period of time. Namely, the inflammation phase of wound healing typically lasts a couple of days and the proliferation phase occurs up to 2–3 weeks after injury [178, 201]. It appears, meanwhile, that the resolution of inflammation is impaired in DR.

Inflammasomes, such as NLRP1, NLRP3, NLRC4, and AIM2, are cytosolic pattern recognition receptors controlling innate immunity [202]. Among them, NLRP3 is the predominant inflammasome activated by tissue injury, contributing to wound healing in the early inflammation phase [203]. As described previously, M2 polarized macrophages produce SPMs, inhibiting the activation of NLRP3 inflammasome [200].

It has been shown that SPMs, such as lipoxin A4 (LXA4) and resolvin D1 (RvD1), possessed highly potent pro-resolving properties [204, 205], inhibiting the activation of NLRP3 inflammasome and promoting the wound healing [206, 207]. However, it was reported that the levels of LXA4 and RvD1 were reduced in diabetic serum [208–211]. Shi et al. demonstrated high glucose induced a decrease in RvD1 levels in the retina of diabetic mouse [208]. Kaviarasan et al. found that a significant decrease in LXA4 levels was observed in PDR vitreous [211]. ALX/FPR2 and GPR32, the receptors for LXA4 and RvD1 [212], were reportedly downregulated by high glucose in the diabetic retina [209, 210]. Therefore, the NLRP3 inflammasome activation observed in PDR is presumably increased by high glucose-induced suppression of SPMs and their receptors, causing chronic inflammation with angiogenesis and fibrosis [213, 214].

It has been proposed that the transition from inflammation to proliferation is a critical step during wound healing [177] and that efferocytosis (removal of dying cells by macrophages) provides a key signal to this transition [215, 216]. Efferocytosis reportedly induces macrophage polarization from pro-inflammatory M1 to anti-inflammatory/pro-angiogenic M2 phenotype [217, 218]. Suresh Babu et al. demonstrated that high glucose conditions induced impairment of efferocytosis in vitro [219]. Khanna et al. found that macrophages isolated from wounds of diabetic mice showed significant impairment in efferocytosis [220].

Freenstra et al. indicated that multiple forms of cell death including apoptosis, necrosis, and pyroptosis were observed in DR [221]. It has been shown that ineffective efferocytosis led to the accumulation of necrotic and pyroptotic cells, releasing DAMPs such as HMGB1 and ATP, which induced vascular permeability [222–225]. DME is frequently sustained without progression to PDR [226], in which high glucose conditions may impair clearance of dying cells by efferocytosis, resulting in the inhibition of macrophage polarization from pro-inflammatory M1 to anti-inflammatory/pro-angiogenic M2 phenotype [217–220]. Thus, it can be concluded that NLRP3 inflammasome activation by downregulation of SPMs and their receptors as well as inefficient efferocytosis under high glucose conditions may cause persistent inflammation in DR.

## Promising new approaches for diabetic retinopathy treatment targeting immunomodulation

As mentioned previously, various immunosuppressants including corticosteroids have been shown to be effective in treating DR, especially DME [23–27]. Since DR appears to have characteristics of an autoimmune disease, it might be worthwhile to investigate other immunosuppressive or immunomodulating therapies for the treatment of DR [227–230].

Orally administered autoantigens suppress autoimmune diseases in animal models, such as collagen-induced arthritis, experimental allergic encephalomyelitis, uveitis, and type I diabetes, by inducing oral tolerance [48, 49, 231]. Autoantigens of these animal models are type II collagen, myelin, S antigen, and insulin, respectively [231]. Low doses of oral antigen induce antigen-specific T cell responses, especially those of regulatory T cells in the gut, releasing anti-inflammatory cytokines including TGF-β, IL-4, and IL-10 [231, 232]. Human trials of orally administered antigen have shown positive findings in patients with RA and MS [231]. As described previously, anti-type II collagen antibodies increase in the serum before DR is clinically manifested [20]; therefore, oral immune tolerance induction with type II collagen could prevent the onset of DR.

As previously mentioned before, it has been suggested that dysregulation of innate immunity associated with increased inflammatory responses contributes to DR progression [134, 135]. It has also been shown that the activation of the NLRP3 inflammasome, a key regulator of innate immunity, may cause the exacerbation of macular edema, angiogenesis, and fibrosis [155, 156, 166–168]. Several NLRP3 inhibitors have been investigated for the treatment of DR [233–235]. Zhang et al. demonstrated that MCC950 had protective effects against high glucose-induced human retinal endothelial cell dysfunction [233]. Trotta et al. observed that β-hydroxybutyrate inhibited diabetic retinal damage through reduction of the NLRP3 inflammasome activation [234]. Isaji et al. reported that tranilast suppressed the proliferation and migration of endothelial cells in vitro and angiogenesis in vivo [235]. As numerous studies have revealed that inhibition of the NLRP3 inflammasome activation was an effective therapeutic approach for autoimmune diseases including RA, MS, and IBD, orally administrable NLRP3 inhibitors such as the abovementioned compounds might be promising candidates for the treatment of DR [236–238].

It has been shown that SPMs (including lipoxins and resolvins) and DHA (the precursor of SPMs) inhibited the NLRP3 inflammasome activity, thus being effective for the prevention of DR [239–243]. Since SPMs have short half-lives [244], epimers and analogs of SPMs might be clinically more useful than SPMs for the treatment of DR [244–246].

## Future issues

**Fig. 4:**
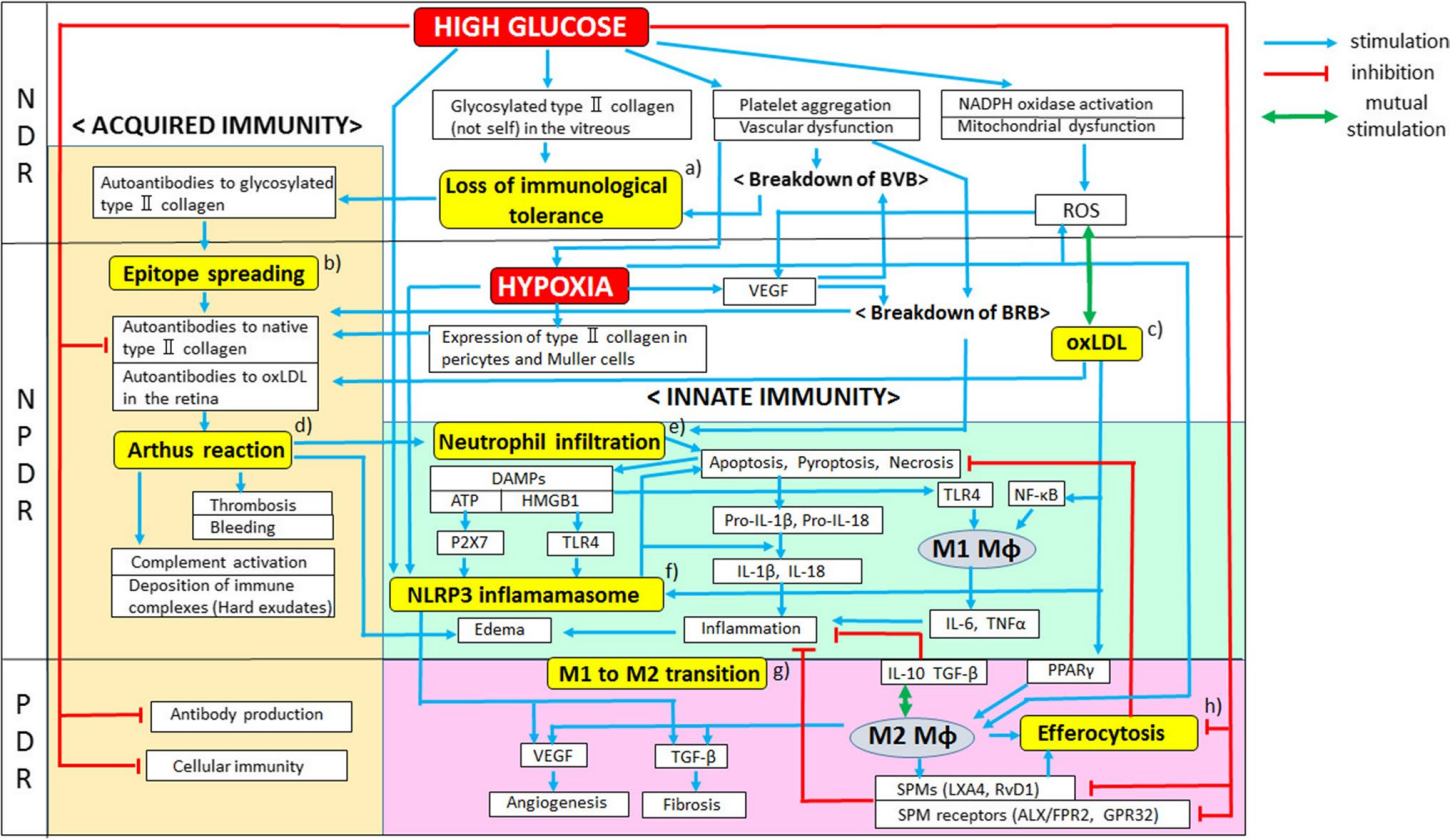
Schematic overview of our hypothesis on the pathogenesis of DR. Acquired immunity (mainly humoral immunity) is presumably involved in the onset of DR, in which glycosylated type II collagen will be recognized as “not self” by T lymphocytes to initiate immune response [78–80], followed by the deposition of immune complexes, causing Arthus reaction, a type of local type III hypersensitivity [33, 87, 88]. Innate immunity enhances the inflammation, angiogenesis, and fibrosis in DR via the activation of the NLRP3 inflammasome, a key mediator of innate immunity and sterile inflammation [134–136]. High glucose suppresses acquired immunity and inhibits the functions of M2 macrophages, resulting in impaired efferocytosis (phagocytosis of dying cells by macrophages) and suppression of SPMs (promoters of the resolution of inflammation) [219, 220], whereas hypoxia induces M2 macrophage polarization via IL-10 and TGF-β [253, 254]. Both high glucose and hypoxia activate innate immunity through NLRP3 inflammasome [134, 135, 164]. **a** High glucose induces glycosylation of type II collagen in the vitreous [80], along with platelet aggregation and vascular dysfunction that may result in the breakdown of BVB, normally sequestering vitreal type II collagen from the immune system [56, 57, 61]. The recognition of glycosylated type II collagen as “not self” by immune cells can cause the loss of immunological tolerance to it [43, 56, 57]. **b** Epitope spreading, generally associated with autoimmune diseases such as RA and MS [255], is defined as an autoimmune response that extends from the initial to additional epitopes within the primary target antigen or from the initial autoantigen to unrelated secondary autoantigens [140–142]. Autoantibodies against native type II collagen, type IV collagen, oxLDL, cardiolipin, and platelet other than glycosylated type II collagen are reportedly observed in the serum of DR patients [14, 17–22]. These multiple autoantibodies are probably generated by epitope spreading. **c** Oxidative stress, such as high glucose and hypoxia, transforms LDL into oxLDL (not self) [256], thus producing autoantibodies and forming immune complexes containing oxLDL in the retina [96]. OxLDL also polarizes macrophages toward the M1 or M2 phenotype via the activation of NF-κB or PPARγ, respectively [257, 258]. Reportedly, low oxidation degree of oxLDL induces M1 macrophages, whereas high oxLDL induces M2 phenotype [259, 260]. **d** RA involves Arthus-type hypersensitivity accompanied with bleeding, thrombosis, edema, neutrophil infiltration, complement activation, and deposition of immune complexes [33, 87, 88]. These clinical findings of RA are also observed in patients with NPDR [19, 93–95], and increased serum levels of autoantibodies to type II collagen are detected in patients with both DR or with RA [20, 31, 32], indicating that these two diseases can have the same etiology. **e** Neutrophil infiltration into the retina is observed in NPDR [10]. The lifespan of infiltrated neutrophils is short and limited by programmed cell death, including apoptosis and pyroptosis (caspase-1-dependent inflammatory cell death) [221, 261], followed by efferocytosis [217–219]. Dying (or dead) neutrophils release inflammatory cytokines (e.g., IL-1β, IL-18) [5] and DAMPs (e.g., HMGB1, ATP) [169, 170], causing retinal inflammation and DME [57, 262]. **f** High glucose, hypoxia, and DAMPs released from dying cells activate the NLRP3 inflammasome in macrophages, resulting in the activation of caspase-1, which cleaves pro-IL-1β and pro-IL-18 into their mature bioactive forms [133]. The activation of the NLRP3 inflammasome also evokes increased levels of VEGF and TGF-β in PDR, promoting angiogenesis and fibrosis, respectively [155, 156, 166]. **g** The development and progression of DR seem to resemble the process of cutaneous wound healing, although their time courses are different. The transition from the inflammatory to proliferative phase is a critical step of wound healing [177]. During the inflammatory phase, neutrophils infiltrate and M1 macrophages produce pro-inflammatory cytokines, whereas, during the proliferative phase, M2 macrophages produce anti-inflammatory cytokines and growth factors, promoting angiogenesis and fibroblast proliferation [184–188]. Inflammatory and proliferative phases of wound healing seemingly correspond to NPDR and PDR, respectively. **h** Efferocytosis provides a key signal to M1 to M2 transition, thus inducing M2 macrophage polarization [217, 218]. M2 macrophages produce SPMs that possesses highly potent pro-resolving properties [199, 200] through inhibiting the activation of NLRP3 inflammasome [206, 207]; however, a high-glucose environment inhibits efferocytosis and SPMs production in DR patients

As described previously, the development and progression of DR appear to resemble cutaneous wound healing. NPDR and inflammation phase of diabetic wound healing, namely the early stages of both diseases, are prolonged, associated with persistent infiltration of neutrophils and with increased permeability of blood vessels [10, 56, 57]. Conversely, the proliferation phase of diabetic wound healing is impaired due to insufficient angiogenesis and inhibition of fibroblast proliferation [247–250], whereas exaggerated angiogenesis and fibrosis occur in PDR (Fig. 4) [38, 39].

In addition, the retina has been considered as an extension of the central nervous system (CNS) anatomically and developmentally [251], and the ocular fundus examination is regarded as the observation of non-invasively visualized CNS in cases of patients with hypertension and atherosclerosis [251, 252]; however, angiogenesis never develops in the brain of diabetic patients. Further study is needed to elucidate what causes the difference of angiogenic activity between PDR and the proliferation phase of diabetic wound healing, and what is the anatomical discrepancy of the blood vessels between the retina and brain in relation to angiogenesis.

## Abbreviations

DR: Diabetic retinopathy
IL: Interleukin
TNF-α: Tumor necrosis factor-alpha
DME: Diabetic macular edema
NPDR: Non-proliferative diabetic retinopathy
PDR: Proliferative diabetic retinopathy
RA: Rheumatoid arthritis
VEGF: Vascular endothelial growth factor
AIED: Autoimmune ear disease
MHC: Major histocompatibility complex
oxLDL: Oxidized low-density lipoprotein
Lp(a): Lipoprotein (a)
LDL: Low-density lipoprotein
MSC: Mesenchymal stem cell
NDR: Non-diabetic retinopathy
NLRP3: NOD-like receptor family pyrin domain-containing 3
DAMPs: Damage-associated molecular patterns
TLR: Toll-like receptor
HMGB1: High-mobility group box 1
ATP: Adenosine triphosphate
IBS: Inflammatory bowel disease
MS: Multiple sclerosis
ROS: Reactive oxygen species
AGEs: Advanced glycation end products
iNOS: Inducible nitric oxide synthase
SPMs: Specialized pro-resolving mediators
LXA4: Lipoxin A4
RvD1: Resolvin D1
BVB: Blood vitreal barrier
BRB: Blood-retinal barrier
MΦ: Macrophage
NF-κB: Nuclear factor κB
PPARγ: Peroxisome proliferator-activated receptor γ
ALX/FPR2: N-formyl peptide receptor-2
GPR32: G protein-coupled receptor 32
TGF-β: Transforming growth factor-beta
TLR4: Toll-like receptor 4
NF-κβ: Nuclear factor κβ
NDR: Non-diabetic retinopathy
MS: Multiple sclerosis
